# Recent Views on Digestion

**Published:** 1870-05

**Authors:** J. R Bromwell


### ARTICLE III.

### *Recent Views on Digestion.*

By J. R Bromwell, D. D. S.

Digestion is the starting point of nutrition—as defined by Dr. Dunglison, t'is a function, by means of which alimentary substances, when introduced into the digestive canal, undergo different alternations, the object of which is to convert them into two parts, the one a reparatory juice, destined to renew the perpetual waste occurring in the economy—the other, deprived of its nutricious properties, to be rejected from the body, or, simply a process, by which the food is reduced to a form in which it can be absorbed from the intestinal canal, and taken up by the blood vessels. Animals alone are furnished with a digestive apparatus. All of them, from man down to the Polypus, contain an alimentary canal or cavity variously shaped. We must therefore conclude, that the existance of a digestive apparatus, is an essential characteristic of the animal kingdom. And that food which is intended to nourish the body, must be subject to a digestive process, before it can be absorbed, and carried through the system. In the vegetable world the case is different, then being no necessity for a process of digestion, owing to the fact that vegetables are dependent upon inorganic substances for their nutrition. Carbonic-acid, saline substances and water, being the chief food of the vegetable kingdom. These materials are constantly supplied to the vegetable, in such a form as to be easily absorbed, existing as they do either in a liquid or gaseous form, therefore, requiring no change, or modification before absorption. After absorption, they are converted into other substances, by the process of nutrition.

With man and animals the case is different, the aliments which nourish them, are obtained, either from the animal or vegetable world, or, as is the case with man, from both. The mineral kingdom furnishes only condiments, medicinal substances, and poisons. Man holds a middle station, between those animals which feed on vegetable and those which feed on animal substances, and he is, therefore, equally fitted for these two kinds of food, being neither herbivorous, nor carnivorous, but in contradistinction is termed omnivorous. The digestive apparatus of the herbivora is more complex than that of the Carnivora, owing to the fact that vegetables are digested with greater difficulty than animal substances. The nutritious matters of vegetables are often present in a solid and unmanageable form, for instance, in common herbs, grass, hay, &c., the digestive matter is so entangled among vegetable cells, fibres &c., and of so small amount, compared to the entire quantity, that a large amount of food must be taken, in order to extract from it the necessary amount of nutritious material, therefore, calling for an extensive and complex digestive process. In such cases, the alimentary canal is long, complex in its structures and functions. In the carnivora the canal is shorter, narrower, and less complicated, the food of such animals being of a softer texture and more easily digested.

With man, though living upon vegetable as well as animal diet, suggesting to us the idea, that his digestive apparatus should resemble to the same extent both the herbivora and the carnivora, it resembles almost exactly that of the carnivora, for most, if not all, of the digestable substances we take as food, are artificially changed and separated from all indigestible impurities, by the process of cooking, and are so softened by the same process, as to be almost, as easily digested as animal substances. Nevertheless, the digestive process of man is still complicated. For as we ascend in the scale of animal existence, we find all the functions of life more complex.

Life is the effect of organization. 'Tis organization in motion. Life is waste, every thonght of our life, every inspiration, every muscular movement, causes the death of some tissue, not the anatomical structures, but the proximate principles, of which they are composed. It has been stated that man wasts daily 1-24 of the weight of his entire body. As this interstitial decay is continually going on, and the body daily, hourly, momentarily wasting, it becomes a matter of critical importance that this waste be supplied, by the formation of new tissue, and it remains for the process of nutrition to supply it. When there is a demand for nutrition, greater than the blood can supply, hunger and appetite may be considered as parts of digestion. In regard to the cause of hunger, many different theories have been propounded, the following, an regarded to be correct. The sensation of hunger, although refered to the stomach, is governed by the system at large, for in proportion as the nutritive material, or property of the blood is sufficient, or inadequate to supply the waste of tissue, sois the sensation of hunger increased or diminished, the want of food being the primary not the proximate cause of hunger, proximate cause of hunger being the waste of tissue and the demand for separation. The sensation of hunger is more keenly felt in the young than in the old, owing to the fact that the constructive and destructive arangements of the young are more active than those of the old, and again, the mind being free from anxiety and not directed towards other objects, takes notice of the sense of hunger at once. Excessive grief and moral emotion deprives us of appetite. Every one is acquainted with the facts, that when laboring under intense mental excitement we have but very little desire for food; the mind being directed in another channel fails to take cognizance of the sensation of hunger, and was it not for the great lassitude and faintness produced by abstinence from food, a person burdened with any great trouble might go for an indefinite period of time without feeling the sensation of hunger, or the slightest desire for food. The effects of protracted abstinence from food are a diminution of the weight of the body, which becomes sensible in the course of twenty four hours, a waste of the body from the loss of fat, discoloration of the fluids—especially the blood, loss of strength, excessive sensibility, sleeplessness, with painful sensations in the epigastric region. Death from inanition is most easily brought on in the young. Thus the unfortunate father, whose horrid story related by Dante, condemned to die of hunger, and shut up with his children in a dark dungeon, died the last on the eighth day, after having witnessed in the convulsions of rage and despair, the death of his four sons, unhappy victums of the most execrable vengence ever recorded in the history of man. There are many marvellous, and if I may be pardoned for saying so, ridiculous accounts on record in regard to the length of time a man may go without food of any kind. Haller, in his work on Physiology, gives an account of a person who went as long as eight years without any food. The average length of time, for a person to go without food, is from five to eight days.

The 10th pair. of nerves, convey the impression of hunger to the brain. The impression of hunger is to the stomach, as the sensation of sleep is to the eyes. Man requires 1-23 of his weight in daily food, to supply the waste of tissue, continually going in his body; if the waste is greater than the repair, we ultimately have death. Hunger kills by a putrid fever; thirst by an inflamatory one. The sensations of thirst seem to be very analygous to those of hunger, but, the seat of sensation of thirst is in the fauces, not in the stomach. The proximate cause of thirst is the demand from the system caused by the discharge of water by the skin, kidneys &c. Primative cause, sensation in the throat. Thirst is in direct ratio to the waste going in the tissues, being increased by high temperatures and loss of blood, also by various kinds of food and drink, for instance salted and highly flavored or seasoned meats, alcoholic and all stimulating drinks, salt producing thirst by the attraction of water from the blood by the process of endosmoses. It is increased in proportion to the increase of the secretions and excreations. In diabetes for instance it is excessive. The calls of thirst are more absolute than those of hunger, and are much less patiently endured. Every one who has witnessed the scene of a battle field after an engagement, will remember the calls of the dying and wounded for water. A drink of cold water to a man suffering from thirst increases his thirst by acting on the coats of the stomach as a stimulant, thereby increasing the flow of blood to the part, and immediately absorbing the water. For fluids taken into the stomach are not subjected to a process of digestion, but are absorbed at once. Tepid drinks are better than cold to satisfy thirst, ice better than water. Three to four days, is the limit of endurance from water, given by most modern physiologists. If the calls of thirst are not satisfied, the blood and the fluids which are formed from it become more and more stimulating, from the concentration of saline substances which they contain. The general irritation gives rise to acute fever, with heat and parching of the fauces, which inflame and may become gangrenous, as happens in the case of hydrophobia. Water constitutes 75 per cent of the human body, and as it is not only given off by the excretions and secretions, but by the waste of tissues also, it is very important that this waste be supplied, showing the necessity, of the free use of cooling drinks in nearly if not all forms of disease. Many a man has languished and died when his life might have been saved by water.

In the foregoing remarks I have endeavered to show the necessity of food and water, not only to the health but to the life of the animal economy.

We now come to the various functions of digestion. In man, the digestive apparatus consists of a tube or canal extending from the mouth to the anus, called the alimentory canal. This canal is separated into various cavities or compartments, communicating with each other by means of narrow orifices. Into these cavities empty the different digestive fluids secreted by the mucous membrane lining the inner surface of the canal, and by the neighboring glandular structures. First we have the cavity of the mouth, communicating with the stomach by the pharynx and aesophagus. Second, the stomach opening into the small intestine by means of the pyloric orifice. Third, the small intestine having the names of duodeum, jejunum and ilium. Fourth, and finally, the large intestine separated from the former by the ileo-coecal valve, and terminating at its lower extremity by the anus guarded by a double sphincter. The alimentary canal is composed of a mucous membrane, an intermediate coat composed of sub-mucous areola-tissue, and a muscular coat. The mucous membrane presents a different structure, and has different properties in different parts of the canal. In the cavity of the mouth, pharynx and æsophagus, it is smooth, with hard, tesselated epithelium. In the stomach this membrane, a continuation of that of the aesophagus, is soft and thick and of a light pink color, it is thrown into a number of folds or rugæ, the epithelium is thinner than that of the æsophagus, and is of the columnar variety. In the small intestine the membrane is covered, with columnar epithelium and longer than in any other coat. It is thrown into numerous transverse folds, called valvular conniventes; its surface is covered by a number of projections call villi, and it contains a variety of peculiar glandular structures. In the large intestine, the membrane is again different, here it is smooth and shining, free from villosities, and has a different glandular structure. The intermediate or cellular coat attaches the mucous to the muscular coat, and serves for the passage of vessels and nerves. The muscular coat is formed throughout its entire length by two layers of fibres, one longitudinal, the other transverse. The alternate contraction, and relaxation of these two sets of fibres produce the peristaltic or vermicular motion, by means of which the food is carried through the canal from above downwards. The will directs, to a certain extent, the motion of its two extremities, while the motion of the rest of the canal, is involuntary. The digestive fluids are also different in different parts of the canal

The food as it passes down the canal meets with no less than five different digestive fluids. First, the saliva, second, gastric juice, third, bile, fourth, pancreatic, fifth, the intestinal juice. Digestion is accomplished throughout the entire canal, by the action of these five different fluids, the various materials of food, are successively reduced to a fluid, transformed and taken up by the absorbent glands, of the mucous membrane of the small intestine.

Digestion is divided into the subordinate functions, of Prehension, Mastication, Insalivation, Deglutition, Gastric digestion, Intestinal digestion, and Defecation.

Prehension is the act of laying hold of, or seizing the food. Mastication is the process, by means of which the food is bruised, ground or triturated, so that it may be readily acted upon by the digestive fluids. It is of great importance to the perfect digestion of food, owing to the fact, that as the action of the digestive fluid is purely chemical in its nature, the food will be more readily acted upon by being thoroughly comminuted than if brought into contact with the fluids in a solid form. In the chemical process of subjecting any body to the solvent action of any fluid, it is always more readily acted upon by being broken into small pieces, presenting a large surface to the contact of the fluid. If mastication is imperfectly effected the subsequent processes of digestion are liable to be deranged. This derangement is continually met with, for there is not, perhaps, a more frequent cause for dyspepsia than imperfect mastication, whether caused by the haste with which the food is swallowed or by the want of proper instruments. The main reason for so many diseases (with man) of the digestive process, is the diseased condition of the mouth and teeth. For food to be digested properly, must be thoroughly mixed with salva, and this cannot be done unless well masticated. Food that has been imperfectly masticated, will be imperfectly digested. Mastication must, therefore, be considered as one of the most important features of digestion. During the mastication of food it is mixed with the first of the digestive fluids, viz. the saliva. Human saliva, as obtained directly from the cavity of the mouth, is a colorless, slightly viscid, and alkaline fluid, with a specific gravity of 1005. Bidder and Schmidt give the following analysis of saliva.

| | |
|---|---|
| Water, . . . . . . . . . . . . . . | 995.16 |
| Organic Matter, . . . . . . . . . | 1.34 |
| Sulph. Cyanide of Potassa, . . . . . . . | 0.06 |
| Phosphates of Soda Lime and Magnesia. . . | .98 |
| Chlorides of Sodium and Potassium, . . . . . | .84 |
| Mixture of Epethelium, . . . . . . . . . . | 1.62 |
| Total | 1000.00 |

The organic substance found in the saliva, is known by the name of Ptyaline. There is a small amount of albumen also present mixed with the ptyaline. The reaction of saliva in a normal condition is decidedly alkaline. Nevertheless, it varies during the day, being at times decidedly acid, this is thought to be owing to the excessive secretion of mucous, the reaction of which is acid. In regard to the quantity of saliva secreted daily, it is almost impossible to form a correct estimate, owing to the fact that the salivary secretions, are by no means constant, being almost, if not entirely, suspended by cessation of the muscles of mastication and the tongue, unless excited by the artificial stimulus of food or some foreign body. Hence it follows, that during sleep, if the mouth be kept open, it become dry because the secretions are checked. The saliva flows readily under the stimulus of food, it is in act proportion to its hardness, or dryness, when dry and hard, therefore, requiring much mastication, then is excessive secretion of saliva. Where soft and moist, a small quantity of saliva is poured out.

Fluids do not excite the secretion of saliva, but pass at once into the stomach. The parotid gland is the principle one, in the secretion of saliva, it assists in mastication. The sub-maxillary, and sub-lingual assists in diglutition. The true function of the saliva is purely mechanical. Its action is to moisten the food, and assist in mastication and deglutition. The saliva has also the property of converting, by a catalytic action, starch into a sugar. This property of saliva is owing to its organic principal ptyaline, which was discovered by Leuchs of Germany. It was fomerly believed that all amylacious substances, were partially, if not wholly, digested in the mouth by the action of the saliva. The present theory in regard to digestion proves the fallacy of the above, owing to the fact that food does not remain long enough in the mouth to be digested, but passes at once, after mastication into the stomach, and also, that the digestion of starch, is accomplished in other parts of the canal to be spoken of hereafter. The action of saliva is therefore simply a physical one.

(*To be continued.*)

